# New Autonomous Water-Enabled Self-Healing Coating Material with Antibacterial-Agent-Releasing Properties

**DOI:** 10.3390/pharmaceutics14051005

**Published:** 2022-05-07

**Authors:** Ki-Hak Kim, Hang-Nga Mai, Dong-Choon Hyun, Du-Hyeong Lee

**Affiliations:** 1 Department of Polymer Science and Engineering, Kyungpook National University, Daegu 41940, Korea; kdi1504@naver.com; 2 Institute for Translational Research in Dentistry, Kyungpook National University, Daegu 41940, Korea; maihangnga1403@knu.ac.kr; 3 Department of Prosthodontics, School of Dentistry, Kyungpook National University, Daegu 41940, Korea

**Keywords:** coating, water-enabled self-healing, antibacterial-releasing, dental restorations, chlorhexidine, tannic acid

## Abstract

A new autonomous water-enabled self-healing coating with antibacterial-agent-releasing capability was developed for the first time by precipitating an aqueous solution of hydrogen-bonded tannic acid (TA) and polyethylene glycol (PEG) (TA: 5 mg/mL; PEG: 5 mg/mL with M_W_ = 100 kDa) to form a smooth, uniform coating layer with an average roughness of 0.688 nm and thickness of 22.3 μm on a polymethyl methacrylate (PMMA) substrate after 10 min of incubation. Our method is cost- and time-efficient, as the hydrophilic coating (water contact angle = 65.1°) forms rapidly, binding strongly to the PMMA substrate (adhesive energy = 83 mJ/m^2^), without the need for pretreatment or surface modification, and is capable of rapid self-repair (approximately 5 min) through hydrogen bonding in aqueous media. Furthermore, adding 0.5 mg/mL of chlorhexidine acetate (CHX), a commonly used antibacterial agent in dentistry, into the TA–PEG emulsion allowed the release of 2.89 μg/mL of the drug from the coating layer, which is promising for actively inhibiting the vitality and growth of bacteria around PMMA dental restorations. The use of CHX-loaded TA–PEG hydrogen-bonded complexes is highly favorable for the fabrication of an autonomous self-healing biocoating with active antibacterial-agent-releasing capability, which can be applied not only in dentistry but also in other medical fields.

## 1. Introduction

Polymethyl methacrylate (PMMA) has been commonly used for several dental applications, including the fabrication of provisional restorations, removable dentures, and obturators, relining, and repair of dental prostheses, due to its ease of manipulation, acceptable esthetics, and high cost-effectiveness [1]. However, the high porosity and water absorption of PMMA make it prone to attachment of oral bacteria, which leads to contamination of the material and failure of the restorations [2]. The application of a protective coating film is one of the most promising strategies to reduce bacterial adhesion and prevent biofilm formation, as the coating does not sacrifice the mechanical properties and integrity of the material [3,4]. Previous research reported coatings that exhibit antiadhesion/bacterial-repelling and contact-killing properties; however, they did not provide antibacterial action beyond the covered surface [4,5,6,7]. Further, the release-killing approach involving the loading and release of antibacterial chemicals, such as antibiotics and antibacterial agents, can actively kill the bacteria before their contact with the substrate surface [8,9].

Once the coating is applied to dental restorations, it must endure mechanical grinding forces generated from chewing and brushing processes, which are unavoidable stressors in the oral environment. Damages, such as surface scratching and microcracking, potentially induce the generation of a microleakage, which can trap oral fluid, promote macroscopic cracking, and eventually lead to the loss of the protective and esthetic functions of the coating [10]. Several polymer-based coatings with self-healing capability have been developed to restore the damages by sealing the primary defects and to prevent further corrosion [10,11]. Generally, self-healing polymeric coatings can be categorized into autonomous healing coating, which is based on the intrinsic physical or chemical repair of the coating matrices, and nonautonomous healing coating, in which healing is induced by the external energy, such as light, heat, or organic solvent stimuli [10]. For watery environments, such as the oral cavity, water-enabled, autonomous self-healing hydrogel coatings are particularly promising because they can be repaired simply by soaking them in water or exposing them to moisture without the use of any special devices or chemicals [12,13]. Furthermore, functional chemicals could be added to the hydrogel coatings to endow them with drug delivery [14] and antifouling [15] properties. Self-healing coatings with antibacterial-agent-releasing properties are very useful to protect dental restorations; however, most of the developed coatings require pretreatment or surface modifications, which are time-consuming and costly for clinical applications [9,16,17,18].

Oral infectious diseases, such as oral candidiasis, gingivitis, periodontitis, dental caries, and peri-implantitis, are detrimental to oral health and, consequently, to systemic health [19]. Bacterial contamination of dental restorations may lead to infections in dental and oral tissues [20]. Inhibition of bacterial growth on dental restorations may reduce the risk of inflammatory responses in the oral tissues. In recent decades, the incorporation of antibacterial drug delivery systems within dental materials, such as dental restorations and implants, has garnered considerable attention [21]. Here, a new autonomous water-enabled self-healing polymer coating with antimicrobial-agent-releasing capability was developed, which can be applied on various dental restorative substrates using a facile, safe, and effective coating protocol. For this purpose, the self-healing hydrogel coating was made by precipitating hydrogen-bonded complexes of tannic acid (TA), a natural polyphenol, and polyethylene glycol (PEG), a biocompatible hydrophilic polyether, in an aqueous solution. After the addition of chlorhexidine acetate (CHX), a commonly used antibacterial agent in dentistry, the CHX-loaded TA–PEG emulsion was coated on the surface of a polymethyl methacrylate (PMMA) substrate to form a uniform coating layer. The CHX-loaded TA–PEG emulsions containing various concentrations of CHX, TA, PEG, and sodium chloride (NaCl) were studied for optimization of coating formation time and coating thickness. Assessments of surface characteristics, mechanical durability, self-healing capability, drug-loading capacity, drug-release behavior, and cell viability and adhesion were further performed to evaluate the effectiveness of the coating.

## 2. Materials and Methods

### 2.1. Materials

TA, CHX, and PEG with various weight-averaged molecular weights (M_W_ = 100 and 200) were purchased from Sigma-Aldrich (St. Louis, MO, USA). NaCl (99.5%) was purchased from Duksan Pure Chemicals Co., Ltd. (Seoul, South Korea). Dulbecco’s Modified Eagle Medium (DMEM) and MC3T3-E1 mouse osteoblast cells were obtained from Gibco (Grand Island, NY, USA) and Korean Cell Line Bank (Seoul, South Korea), respectively. All the reagents were used as received without further purification. Deionized (DI) water with a resistivity of 18.2 MΩ cm, produced by a water ultrapurification system (HIQ, Human Science, Gyeonggi-do, South Korea), was used as a medium for the preparation of coating layers.

### 2.2. Preparation of CHX-Loaded TA–PEG Emulsions

**Figure 1:**
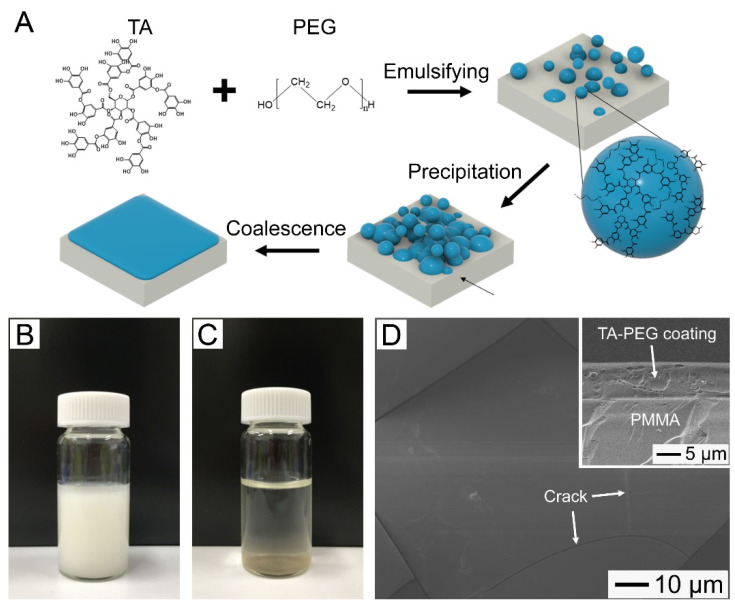
(**A**) Schematic illustration of the procedure for preparing an antibacterial coating layer with self-healing and drug-releasing capabilities. (**B**,**C**) Photographs of the TA-PEG emulsion containing NaCl (1 M) taken: (**B**) Immediately and (**C**) at 60 min after mixing TA (10 mg/mL) and PEG (2 mg/mL, M_W_ = 100 kDa) aqueous solutions. (**D**) SEM image of the PMMA substrate incubated with the emulsion of (**B**) for 120 min. The inset shows a cross-sectional SEM image of the substrate.

The procedure for the fabrication of the CHX-loaded TA–PEG coating is schematized in Figure 1A. First, a white emulsion consisting of TA–PEG complexes was prepared by mixing an aqueous solution of TA (10 mL) and an aqueous solution of PEG (10 mL) under mechanical agitation (Figure 1B). When NaCl was added to the mixture, precipitation of the emulsion occurred (Figure 1C). In the preparation of the CHX-loaded TA–PEG emulsions, different concentrations of CHX (0.1, 0.3, and 0.5 mg/mL) were added to the TA–PEG emulsion containing 7.5 mg/mL TA, 5 mg/mL PEG with M_W_ = 100 kDa, and 2-M NaCl.

To study the effects of TA/PEG ratio and PEG molecular weight on the coating formation, we fabricated different TA–PEG emulsions containing various concentrations of TA, PEG, and NaCl (2.5, 5, 7.5, and 10 mg/mL of TA; 0.5, 1, 2.5, and 5 mg/mL of PEG with M_W_ = 100 and 200 kDa; and 1 and 2 M of NaCl). To elucidate the influence of NaCl on the formation of the TA–PEG coating layer, the zeta potential values of the emulsions with and without the salt were measured using Zetasizer Nano ZS (Malvern Instruments, Worcestershire, UK).

### 2.3. Coating Procedure

Following the manufacturer’s instructions, 40 square-shaped resin specimens (1.5 cm × 1.5 cm, 1 mm in thickness) were fabricated using PMMA for dental resin restorations (ALIKE, GC America, Alsip, IL, USA). The specimens were prewashed with DI water, placed on the bottom of a polystyrene Petri dish (diameter 100 mm, height 15 mm), and immersed in the CHX-loaded TA–PEG emulsion to form a uniform coating layer. The coated specimens were then taken out from the Petri dish, stored under ambient condition for 1 h, rinsed with DI water, and completely dried in air at room temperature. To systematically investigate the coating formation behavior through precipitation, we monitored the change in the surface morphologies of the PMMA substrates with the emulsion containing 5 mg/mL of TA, 1 mg/mL of PEG with M_W_ = 100 kDa, and 1 M of NaCl during different incubation periods (10, 20, 40, and 90 min).

### 2.4. Investigation of the Surface Characteristics and Mechanical Durability of the Coated Specimens

The surface morphologies of the coated specimens were investigated by scanning electron microscopy (SEM, SU-8220, Hitachi, Tokyo, Japan) and atomic force microscopy (AFM, NX20, Park Systems, Suwon, South Korea). To examine the surface tension of the TA–PEG coating layer, we measured the contact angles of DI water and diiodomethane using a contact angle measurement system (DSA100, KRÜSS GmbH, Hamburg, Germany). Based on the contact angle values, the surface tension of each TA–PEG coating layer (*γ*_TA__–PEG_) could be calculated. In the calculation, we used the Owens–Wendt model [22]:

*γ*_L_(1 + cos *θ*) = 2[(*γ*_L_^D^ × *γ*_TA–PEG_^D^)^1/2^ + (*γ*_L_^P^ × *γ*_TA–PEG_^P^)^1/2^] (1)

where *γ*_L_ (*γ*_L_ = *γ*_L_^P^ + *γ*_L_^D^) denotes the surface tension of the test liquid, DI water (*γ*_Water_ = 72.8 mN/m, *γ*_Water_^P^ = 51 mN/m, *γ*_Water_^D^ = 21.8 mN/m) and diiodomethane (*γ*_Diiodomethane_ = 50.8 mN/m, *γ*_Diiodomethane_^P^ = 0 mN/m, *γ*_Diiodomethane_^D^ = 50.8 mN/m); *γ*_L_^P^ and *γ*_L_^D^ denote the polar and dispersion components of *γ*_L_; *γ*_TA–PEG_^P^ and *γ*_TA–PEG_^D^ denote the polar and dispersion components of *γ*_TA__–PEG_; and *θ* denotes the contact angle of each test liquid.

To further study the mechanical durability of the coating layer, its hardness was measured by employing the Wolff–Wilborn method (pencil hardness test) using pencils with hardness grades ranging from 9B to 9H under a loading force of 7.5 N.

### 2.5. Assessment of Self-Healing Capability

To investigate the self-healing performance of the coating layer, a cut with a width of approximately 50 μm on the surface of each coated sample was made deep enough to expose the surface of the underlying PMMA substrate. Then, the sample was incubated with water for different time periods (0, 1, 5, and 10 min). In situ observation of the healing process was performed by monitoring the change in the surface morphology of the sample by optical microscopy (BX51, Olympus, Tokyo, Japan).

### 2.6. Assessment of the Drug Encapsulation Efficiency and Drug-Release Behavior

To examine the actual amount of loaded CHX in the coating layer, the CHX-loaded/TA-PEG coating was completely dissolved in aqueous NaOH (3 mL, 0.01 M), and the resultant solution was transferred to a dialysis tube with a molecular weight cutoff of 2 kDa. The tube was then placed in the corresponding aqueous solution (47 mL). After 5 days, 40 mL of the solution was removed, followed by freeze-drying. Finally, 3 mL of DI water was added for high-performance liquid chromatography (HPLC) characterization (LC-20AT prominence system, Prominence, Shimadzu, Japan).

To investigate the release behavior of CHX from the coating layer, the coated specimens and 3 mL of phosphate-buffered saline (PBS) solution (pH 7.4) were placed in a dialysis tube with a molecular weight cutoff of 2 kDa. Afterward, the tube was placed in PBS solution (47 mL) at 37 °C. At determined time points, 40 mL of the solution was taken out, followed by replenishing 40 mL of fresh PBS solution. Then, the taken solution was freeze-dried, and 3 mL of DI water was introduced to obtain a concentrated solution of CHX, which was used for HPLC characterization.

For the HPLC characterization, chromatographic separation was carried out on Discovery C18 columns (150 mm × 4.6 mm, 5 μm, Supelco, Bellefonte, PA, USA) at 30 °C using a solvent mixture, consisting of an aqueous solution of trifluoroacetic acid (0.1 vol%) and acetonitrile at a ratio of 3:2 in volume, as the mobile phase. The injection volume was 10 μL, and the flow rate was 1.0 mL/min for a running time of 10 min. Detection was performed by absorbance of UV light at a wavelength (λ) of 254 nm. The amount of released CHX was determined using a calibration curve obtained from a series of CHX (5, 10, 20, 50, 70, and 100 μg/mL).

### 2.7. Cell Viability and Cell Adhesion Tests

The cell viability and cell adhesion tests of the TA–PEG coating layers were performed by subjecting MC3T3-E1 cells to a live/dead assay. Briefly, the PMMA substrate coated with TA–PEG was placed in a 12-well plate, followed by seeding of MC3T3-E1 cells at a density of 5 × 10^4^ cells/well and culturing with DMEM at 37 °C in an atmosphere of 5% CO_2_ for 1 day. Then, the medium was removed and the sample was washed twice with PBS. A live/dead assay was then performed using a commercially available kit (Biotium, Fremont, CA, USA) [23]. Fluorescence induced by the assay reagents was monitored using a confocal laser scanning (CLS) microscope (LSM700, Carl Zeiss, Oberkochen, Germany).

## 3. Results

### 3.1. Coating Layer Formation on the PMMA Substrate

The formation of the TA–PEG coating layer is shown in Figure 1. The average value of the water contact angle of the treated PMMA specimens (65.1°) was smaller than that of the untreated PMMA specimens (75.2°) (Figure S1). The decrease in the contact angle suggests the formation of a relatively hydrophilic coating on the substrate. The decrease in the contact angle suggests that relatively hydrophilic TA-PEG complexes were coated on the substrate. This coating can be visualized after subjecting the sample to mechanical stress. As shown in Figure 1D, sharp cracks were observed, indicating the formation of the TA-PEG coating layer more directly. The cross-sectional SEM image in the inset demonstrates that the layer was uniform in thickness (7.8 μm on average). Furthermore, delamination of the coating layer was not observed, indicating its good binding to the substrate, as a result of the substrate-independent surface anchoring capabilities of TA (imparted by the dihydroxyphenyl and trihydroxyphenyl groups present in the molecule) [24,25,26].

A key to the successful formation of the TA-PEG coating layer is to induce the precipitation of TA-PEG complexes, which could be achieved by the addition of NaCl to the emulsion. The zeta potential of the emulsion (5 mg/mL of TA, 1 mg/mL of PEG with M_W_ = 100 kDa) in the absence of NaCl was −26.8 mV, which was large enough to stabilize the emulsion by electrostatic repulsion. Consequently, the emulsion was very stable up to 2 days without any precipitation, resulting in no change in water contact angle on the incubated PMMA substrate (Figure S2). However, in the presence of 1 M NaCl, the zeta potential increased to −4.7 mV (Figure S3). This result indicates that Na^+^ ions in the salt screened the negative charge of the TA–PEG complexes, leading to increased coagulation of the complexes, consequently inducing their gravitational precipitation.

### 3.2. Optimization of Coating Formation Time and Coating Thickness

**Figure 2:**
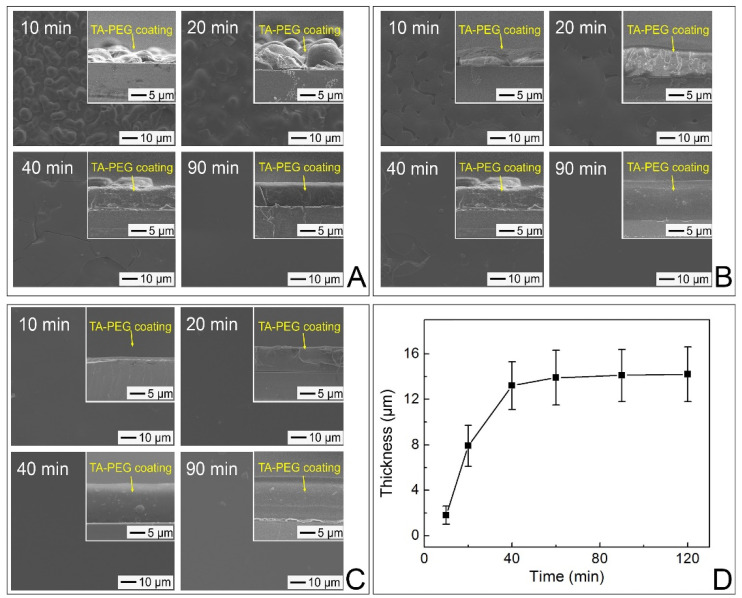
(**A**) 5 mg/mL of TA, 1 mg/mL of PEG with M_W_ = 100 kDa, and 1 M NaCl; (**B**) 5 mg/mL of TA, 2.5 mg/mL of PEG with M_W_ = 100 kDa, and 1 M NaCl; (**C**) 5 mg/mL of TA, 2.5 mg/mL of PEG with M_W_ = 100 kDa, and 2 M NaCl for different periods (10 min, 20 min, 40 min, and 90 min). The cross-sectional SEM images are shown in the insets. (**D**) Changes in thickness of the coating layers on the surface of PMMA substrates incubated with the TA–PEG emulsion containing 5 mg/mL of TA, 2.5 mg/mL of PEG of 100 kDa, and 2 M NaCl.

The changes in the surface morphologies of the PMMA substrates during their incubation with the TA–PEG emulsion (5 mg/mL TA; 1 mg/mL PEG with M_W_ = 100 kDa; 1 M NaCl) are shown in Figure 2A. Incubation for 10 min allowed partial coating with the TA–PEG complexes due to the initial precipitation of the spherical complexes. As incubation further proceeded, more complexes precipitated and then coalesced each other, thereby increasing the coating coverage. After 90 min of incubation, a TA–PEG coating layer with a smooth surface was formed. Its average thickness as determined from the cross-sectional SEM image (inset) was approximately 7.6 μm, which was similar to the thickness of the sample incubated for 120 min. When a higher amount of PEG (2.5 mg/mL PEG with M_W_ = 100 kDa) was used (Figure 2B), a larger coating coverage was observed after 10 min of incubation. A uniform coating layer with an average thickness of 13.2 μm was formed after 60 min of incubation, and further incubation did not change the surface morphology and thickness (Figure S4).

A faster coating formation was observed when the emulsion was prepared with a higher amount of NaCl (5 mg/mL TA; 2.5 mg/mL PEG with M_W_ = 100 kDa; 2 M NaCl). A uniform layer with an average thickness of 1.8 μm was formed on the PMMA substrate after 10 min of incubation (Figure 2C). Its root means square (RMS) roughness as measured by AFM was 0.688 nm, suggesting that its surface was very smooth. The incubation for 20 and 40 min increased the thickness of the coating layer to 7.9 and 13.2 μm (Figure 2D). However, after 60 min, there was no increase in thickness because precipitation was completed.

**Figure 3:**
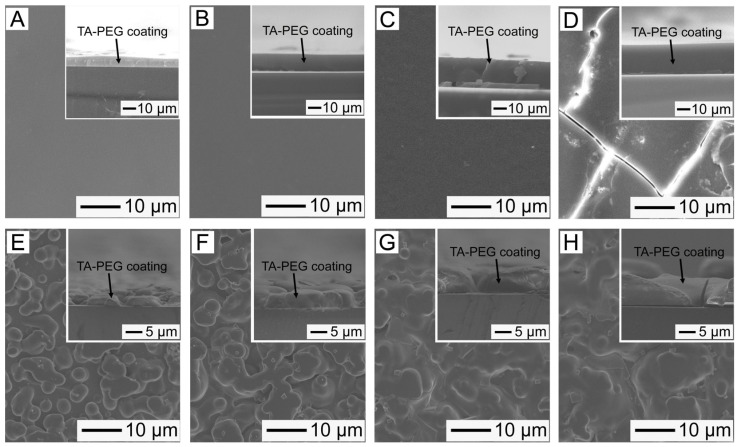
(**A**–**D**) SEM images of the surface morphologies of the PMMA substrates incubated for 10 min with the TA–PEG emulsions (5 mg/mL PEG with M_W_ = 100 kDa; 2 M NaCl) containing different concentrations of TA: (**A**) 2.5 mg/mL, (**B**) 5.0 mg/mL, (**C**) 7.5 mg/mL, and (**D**) 10 mg/mL. (**E**–**H**) SEM images of the surface morphologies of the PMMA substrates incubated for 10 min with the TA–PEG emulsions (0.5 mg/mL PEG with M_W_ = 200 kDa; 2 M NaCl) containing different concentrations of TA: (**E**) 2.5 mg/mL, (**F**) 5 mg/mL, (**G**) 7.5 mg/mL, and (**H**) 10 mg/mL. The cross-sectional SEM images are shown in the insets.

Figure 3A–D shows the SEM images of the PMMA substrates obtained after incubation for 10 min with the emulsion (5 mg/mL PEG with M_W_ = 100 kDa, and 2 M NaCl) containing 2.5, 5.0, 7.5, and 10 mg/mL of TA, respectively. The thickness of the coating layer was increased from 7.4 μm to 22.6 μm when the amount of TA increased from 2.5 mg/mL to 7.5 mg/mL. Smooth surfaces were observed in all the samples, which were also confirmed by their small RMS roughness values (Figure S5). However, the sample obtained from the emulsion containing 10 mg/mL of TA exhibited many macroscopic cracks on its surface (Figure 3D). Figure 3E–H shows the SEM images of the PMMA substrates after incubation for 10 min with the emulsions (PEG with M_W_ = 200 kDa, 0.5 mg/mL; NaCl, 2 M) containing different amounts of TA. Rough surfaces were observed in all samples, and their thickness measurements were smaller compared with those of the samples shown in Figure 3A–D.

### 3.3. Surface Characteristics and Mechanical Durability of the Coated PMMA Specimens

**Figure 4:**
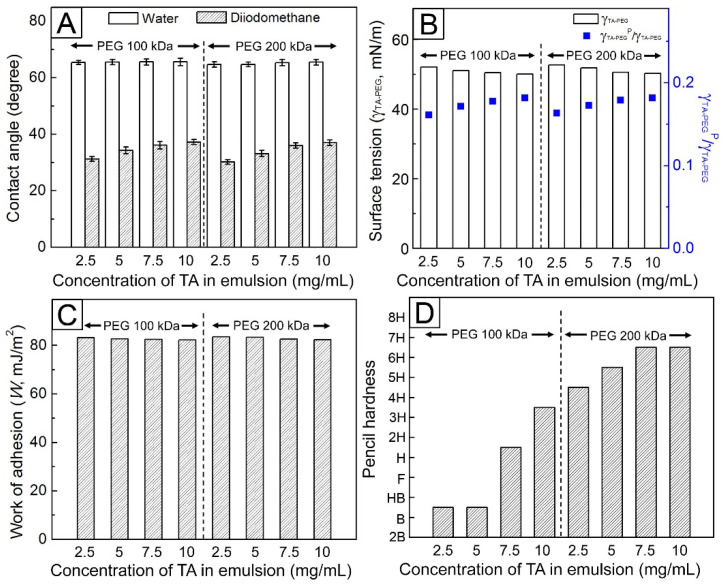
(**A**) Contact angles of water and diiodomethane droplets on the coating layers made using the TA–PEG emulsions containing different concentrations of TA. (**B**) Surface tension (γ_TA-PEG_) and polar contribution (γ_TA-PEG_^P^/γ_TA-PEG_) of coating layers made using the TA–PEG emulsions containing different concentrations of TA. (**C**) Work of adhesion between the TA–PEG coating layer and PMMA substrate in the samples. (**D**) Hardness of the TA–PEG coating layers made using the TA–PEG emulsions containing different concentrations of TA.

For the layers prepared using PEG with M_W_ = 100 kDa, there was no significant difference in water contact angles, whereas the contact angles of diiodomethane increased with the addition of more TA (Figure 4A). A similar trend was observed in the samples prepared from PEG with M_W_ = 200 kDa. Figure 4B shows the calculated *γ*_TA__–PEG_ for each system, which demonstrates that *γ*_TA__–PEG_ slightly decreased with the addition of more TA in the emulsion irrespective of the PEG molecular weight. The polar contribution in the surface tension (*γ*_TA__–PEG_^P^/*γ*_TA__–PEG_) was stronger in the sample made from the emulsion containing more TA. Using these results and the mathematical equation for estimating the work of adhesion [27], the adhesive energy (W) between the TA–PEG coating layer and PMMA substrate in each system could be determined. A similar work of adhesion value, approximately 83 mJ/m^2^, was obtained for all samples (Figure 4C).

The results of the pencil hardness test with respect to the TA–PEG layers are shown in Figure 4D. The use of emulsions with a higher TA content allowed for the fabrication of a TA–PEG coating layer with increased hardness. For example, the sample prepared from an emulsion containing 5 mg/mL PEG (M_W_ = 100 kDa) and 10 mg/mL TA did not exhibit any scratches in the 3H pencil hardness test, whereas a deep scratch from an HB pencil appeared on the surface of the sample prepared using an emulsion containing 5 mg/mL PEG (M_W_ = 100 kDa) and 2.5 mg/mL TA. The PEG molecular weight likewise affected the hardness of the coating layer. When the same concentration of TA was used, increasing the molecular weight of PEG resulted in the TA–PEG coating layer becoming harder. In the 4H pencil hardness test, a clear pencil mark remained on the surface of the TA–PEG coating layer obtained from the emulsion containing 0.5 mg/mL PEG (M_W_ = 200 kDa) and 2.5 mg/mL TA, though scratches were absent, indicating that the hardness of this coating layer was higher than that of the layer containing 5 mg/mL PEG (M_W_ = 100 kDa) and 2.5 mg/mL TA. These differences arise from stronger hydrogen bonds attributed to the presence of a greater number of hydrogen bond donors and acceptors in the TA molecules and PEG chains, respectively.

### 3.4. Self-Healing Capability of the Coating Layers

**Figure 5:**
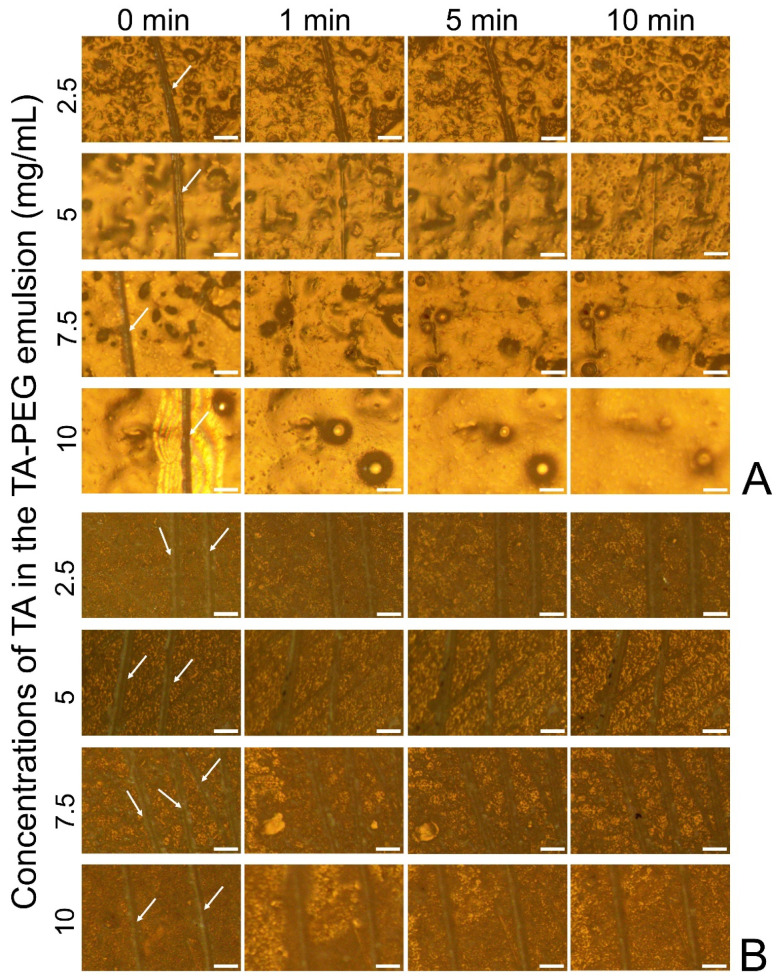
Optical micrographs showing the water-enabled healing behavior of the damaged coating layers obtained in 0 min, 1 min, 5 min, and 10 min from: (**A**) TA–PEG emulsions (5 mg/mL PEG with M_W_ = 100 kDa; 2 M NaCl) containing different concentrations of TA (2.5 mg/mL, 5 mg/mL, 7.5 mg/mL, and 10 mg/mL). (**B**) TA–PEG emulsions (0.5 mg/mL PEG with M_W_ = 200 kDa; 2 M NaCl) containing different concentrations of TA (2.5 mg/mL, 5 mg/mL, 7.5 mg/mL, and 10 mg/mL). The damages formed on the samples are marked by arrows. Scale bars represent 200 μm.

The self-healing capability of the coating layer was shown in Figure 5. The coated specimens made from the emulsions containing PEG with M_W_ = 100 kDa self-repaired the damages in an average of 5 min of immersion in water. The samples containing more TA completed the autonomous healing faster due to its advantage in the reconstruction of hydrogen bonds (Figure 5A). For the samples prepared using PEG with M_W_ = 200 kDa, the repair did not occur, as shown in Figure 5B.

### 3.5. Antibacterial Agent Encapsulation Efficiency and Release Capability of the TA–PEG Coatings

The drug encapsulation efficiency, defined as the ratio of the amount of loaded drug to the amount of drug initially administered and expressed as a percentage, was 1.9%. This low encapsulation efficiency resulted from the use of a relatively small substrate (1.5 cm × 1.5 cm, 1 mm in thickness). As described in the coating procedure, each PMMA substrate was placed at the bottom of a polystyrene Petri dish (diameter, 10 cm; height, 15 mm), followed by immersion in the CHX-loaded TA–PEG emulsion. Taking into consideration the sizes of the substrate and Petri dish, only 2.8% of the precipitated complexes were placed on the substrate. When a PMMA substrate with a diameter of 10 cm was used, the encapsulation efficiency increased to 68.2%, which is comparable to the theoretical value (66.2%).

**Figure 6:**
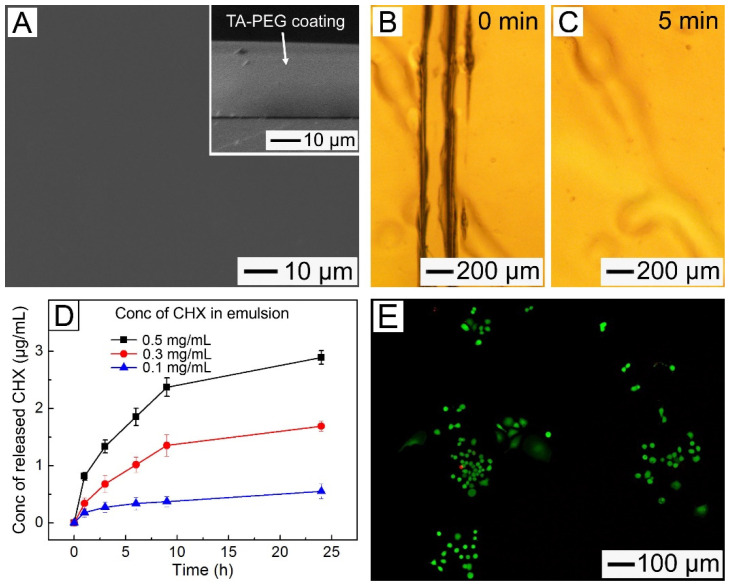
(**A**) SEM image of the PMMA substrate coated with the CHX-loaded TA–PEG coating layer containing 5 mg/mL TA, 5 mg/mL PEG (with M_W_ = 100 kDa), 2 M NaCl and 0.1 mg/mL CHX for 10 min. The inset shows a cross-sectional SEM image of the substrate. (**B**,**C**) Optical micrographs of the CHX-loaded TA–PEG coating layer with a cut on its surface: (**B**) before and (**C**) after exposure to water for 5 min. (**D**) Release profiles of CHX from the TA–PEG coating layers prepared using the emulsions with different concentrations of the drug. (**E**) Confocal laser scanning (CLS) micrograph showing live (green) and dead (red) MC3T3-E1 cells on the TA–PEG layer prepared using the emulsion containing 7.5 mg/mL TA, 5 mg/mL PEG (M_W_ = 100 kDa), and 2 M NaCl.

The SEM image of the resultant sample after immersing a PMMA substrate in the TA–PEG emulsion (5 mg/mL TA; 5 mg/mL PEG with M_W_ = 100 kDa; 2 M NaCl) containing 0.1 mg/mL CHX for 10 min shows the formation of a 22.3 μm thick TA–PEG layer with a smooth surface, which contains 37.6 μg of CHX (Figure 6A). The sample exhibited a rapid self-healing ability after contact with water (Figure 6B,C). The release profile of CHX in PBS buffer at 37 °C from the coated sample (Figure 6D) indicated that the released drug concentration could be higher by increasing the amount of CHX loaded in the coating layer. Herein, when 0.3 and 0.5 mg/mL of CHX were added into the TA–PEG emulsion, the formed coating layer had a CHX loading of 108.2 μg and 183.2 μg and released 1.69 μg/mL and 2.89 μg/mL of the drug, respectively, over the same period.

### 3.6. Cell Viability and Cell Adhesion

The cell viability and cell adhesion of the TA–PEG coating layer are shown in Figure 6E. The calcein-mediated bright green fluorescence produced by live cells was observed, suggesting excellent biocompatibility of the coating layer. Furthermore, the density of the MC3T3-E1 mouse osteoblast cells on the surface of the TA–PEG-coated substrate was considerably lower than that on the surface of the bare PMMA substrate (Figure S6), suggesting that the coating was resistant to fouling by the cells. The nontoxicity and cell fouling resistance of the TA–PEG coating layer, along with its rapid fabrication, self-healing, and antibacterial-agent-releasing properties, suggest its significant potential for dental restoration applications.

## 4. Discussion

In this study, a new CHX-loaded TA–PEG coating material for the PMMA substrates was developed. To find the optimal formula of the TA–PEG emulsion, we investigated the formation time and thickness of the coatings fabricated with TA–PEG emulsions containing various concentrations of TA, PEG, and NaCl. The experimental results indicated that the use of more PEG and the addition of NaCl to the emulsion was effective to induce faster coating formation. This rapid coating formation was due to the stronger charge screening obtained by adding Na+, which facilitated the precipitation of the TA–PEG complexes. Although we succeeded in achieving a uniform coating using the TA–PEG complexes after 10 min of incubation, the formed layer was relatively thin, which is disadvantageous in self-healing, particularly in compensation of the material lost from the coating by severe damage [15,28].

To achieve a thicker coating layer in 10 min, the emulsion was prepared with 10 mg/mL of PEG (M_W_ = 100 kDa) and 2 M of NaCl. Additionally, TA–PEG coating layers with different thicknesses were fabricated by varying the amount of TA in the emulsion. The results show that although the use of higher amounts of TA increased the thickness of the formed layer, some defects were observed on the surfaces of the samples coated with the emulsion containing 10 mg/mL of TA. These defects were generated because of the increase in the number of hydrogen-bonding donors, which are dihydroxyphenyl and trihydroxyphenyl groups present in TA [29,30,31]. The extensive cross-linking in the TA–PEG coating layer makes the layer fragile and consequently form cracks due to volume contraction during drying. The results of water and diiodomethaneon contact angle measurements indicated that the surface tension of each TA–PEG coating layer (*γ*_TA__–PEG_) was slightly decreased with the addition of more TA in the emulsion irrespective of the PEG molecular weight.

Based on the surface characteristics of the coated specimens, the coating layer prepared using emulsions containing 200 kDa PEG exhibited a rough surface. To investigate this, we measured the hydrodynamic diameters of the emulsions prepared using 5 mg/mL PEG (M_W_ = 100 kDa) (shown in Figure 3A) and 0.5 mg/mL PEG (M_W_ = 200 kDa) (shown in Figure 3E) employing a Zetasizer Nano ZS (Malvern Instruments). The former exhibited a hydrodynamic diameter of 107 nm, whereas the latter exhibited a hydrodynamic diameter of 543 nm. This result indicates that PEG with a larger molecular weight generated a larger TA-PEG emulsion because of stronger hydrogen bonds between the polymer and TA. Furthermore, the stronger interaction impeded coalescence between the precipitated complexes, leading to the formation of a coating layer with a rough surface.

Once a coated dental restoration is placed in the oral cavity, the coating layer should endure the mechanical grinding forces generated from chewing and brushing processes. In our study, the TA–PEG coating layer exhibited autonomous self-healing capability to increase its length of service due to the rebuilding of hydrogen bonds between the polymer and TA. When the damaged coating layer is exposed to water, the weakened hydrogen bonding between TA and PEG makes the layer slightly fluidic to fill the damage and thus heal it through the reconstruction of hydrogen bonds [12]. However, the time required for the repair depended on the amount of TA. The addition of a higher amount of TA may increase the healing speed due to its advantage in the reconstruction of hydrogen bonds. In the meanwhile, the higher molecular weight of PEG (M_W_ = 200 kDa) may obstruct the repairing process, which resulted from the slow dynamics of longer polymer chains.

In this study, the coating layer was fabricated with dual functionality of self-healing and antibacterial-agent-releasing capability. For this purpose, the CHX was added to the TA–PEG emulsion. As a result, the CHX-released amount was higher than its minimum inhibitory concentration for clinical isolates of Streptococcus mutans, which was reported to range from 0.25 to 1 µg/mL [32,33]. The incorporation of the drug molecules into the coating layer did not affect its formation and self-healing properties. A higher concentration of drug release could be obtained by increasing the CHX loading in the coating layer. Additionally, the addition of drug delivery carriers, such as liposomes or polymer nanoparticles, could increase the drug-loading capacity and control the drug-releasing rate that contributes to the maintenance of a stable and long-term antibacterial effect [9,34].

The formation of dental plaque, which is a typical biofilm, begins with the adhesion of bacteria or cells that recognize proteins adsorbed onto the dental surface [35,36,37]. To prevent dental biofilm formation, numerous studies have focused on the inhibition of protein adsorption onto dental surfaces. To this end, PEG-based materials have been used to inhibit fouling as PEG effectively inhibits nonspecific protein adsorption [38,39]. The results of the cell viability and adhesion tests in the present study indicated that the coating layer could effectively suppress protein adsorption owing to the inclusion of PEG, resulting in resistance to cell adsorption. However, because the oral environment is complex and dental plaque generation depends on a number of factors, the inhibition of dental biofilm formation on the TA–PEG coating and its stability and bacterial viability in the oral environment need to be investigated in vivo.

In this study, we succeeded in developing a new water-enabled self-healing coating material with several promising features. However, this study is not without limitations. First, the differences between this in vitro experimental study and the complex human oral environment could affect translatability. Second, the release profile and cell viability were evaluated within a short duration. Therefore, to substantiate the impact of this study, comprehensive in vivo and clinical studies with long-term follow-up are necessary. Additionally, the compatibility of the coating with various dental materials and the effects of the coating on different types of bacteria should be investigated to expand the application of this new coating method.

## 5. Conclusions

In summary, a new water-enabled self-healing coating with antimicrobial-agent-releasing capability was successfully fabricated via a rapid, facile, yet effective, method. In particular, the use of the emulsion containing 5 mg/mL of TA, 5 mg/mL of PEG with M_W_ = 100 kDa, and 2 M of NaCl led to the formation of a uniform coating layer with an average thickness of 22.3 μm on the PMMA substrate in 10 min. The addition of 0.5 mg/mL CHX into the emulsion allowed the release of up to 2.89 μg/mL of the drug from the coating layer. The rapid fabrication, self-healing, and antibacterial-agent-releasing properties of the coating layer suggest its great potential in protecting dental restorations.

## Data Availability

The data presented in this study are contained within the article and provided supplementary materials.

## Supplementary Materials

The following supporting information can be downloaded at: https://www.mdpi.com/article/10.3390/pharmaceutics14051005/s1, Figure S1: Water droplets on: (A) the pristine PMMA substrate and (B) the PMMA substrate incubated with the TA–PEG emulsion (5 mg/mL TA, 1 mg/mL PEG of M_W_ = 100 kDa, 1 M NaCl) for 120 min; Figure S2: (A–B) Photographs of the TA-PEG emulsion without the inclusion of NaCl, which were taken: (A) immediately and (B) at 2 days after mixing TA (10 mg/mL) and PEG (2 mg/mL, Mw = 100 kDa) aqueous solutions. (C) Water droplet on the PMMA substrate incubated with the emulsion in (A) for 120 min; Figure S3: Zeta potential of the emulsions with and without NaCl (1 M); Figure S4: SEM images of the surface morphologies of the PMMA substrates incubated with the TA-PEG emulsion (TA: 5 mg/mL, PEG of M_W_ = 100 kDa: 2.5 mg/mL, NaCl: 1 M) for 120 min. The inset shows a cross-sectional SEM image of the substrate. The inset shows a cross-sectional SEM image of the substrate; Figure S5: AFM images showing the surface roughness of the PMMA substrate, which were prepared through incubation for 10 min, with the TA-PEG emulsions (PEG of M_W_ = 100 kDa: 5 mg/mL, NaCl: 2 M) containing different concentrations of TA: (A) 2.5 mg/mL, (B) 5.0 mg/mL, (C) 7.5 mg/mL, and (D) 10 mg/mL; Figure S6: Confocal laser scanning (CLS) micrograph images showing live (green) and dead (red) MC3T3-E1 mouse osteoblast cells on the surface of (A) PMMA bare substrate and (B) TA-PEG-coated substrate.
