# Supplementary material for: New Autonomous Water-Enabled Self-Healing Coating Material with Antibacterial-Agent-Releasing Properties
Source: Pharmaceutics. 2022 May 7;14(5):1005. doi: 10.3390/pharmaceutics14051005 (PMC9143542; doi:10.3390/pharmaceutics14051005)
Supplement: Supplementary file 1 [file pharmaceutics-14-01005-s001.zip › pharmaceutics-1662845-supplementary.pdf]

pharmaceutics

Supplementary Materials: New Autonomous Water-Enabled Self-Healing Coating Material with Antibacterial-Agent Release Property

Ki-Hak Kim, Hang-Nga Mai, Dong-Choon Hyun and Du-Hyeong Lee

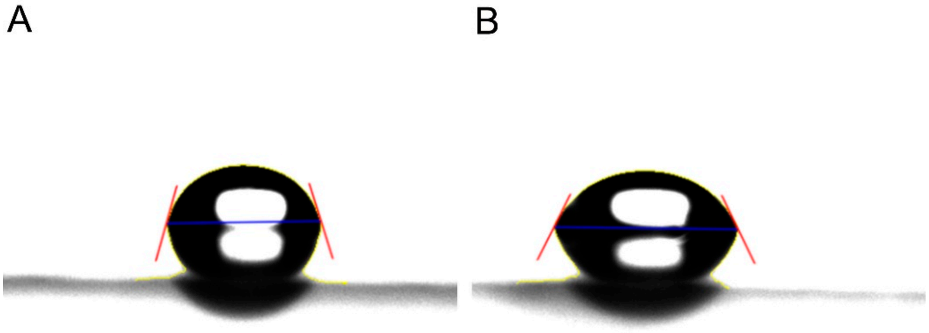

Figure S1. Water droplets on: (**A**) the pristine PMMA substrate and (**B**) the PMMA substrate incubated with the TA–PEG emulsion (5 mg/mL TA, 1 mg/mL PEG with M_W = 100 kDa, 1 M NaCl) for 120 min.

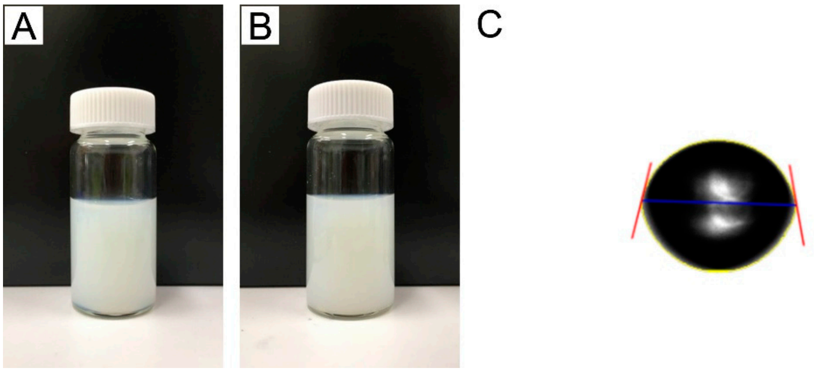

Figure S2. **(A,B)** Photographs of the TA-PEG emulsion without the inclusion of NaCl, which were taken: **(A)** immediately and **(B)** at 2 days after mixing TA (10 mg/mL) and PEG (2 mg/mL, M_w = 100 kDa) aqueous solutions. **(C)** Water droplet on the PMMA substrate incubated with the emulsion in **(A)** for 120 min.

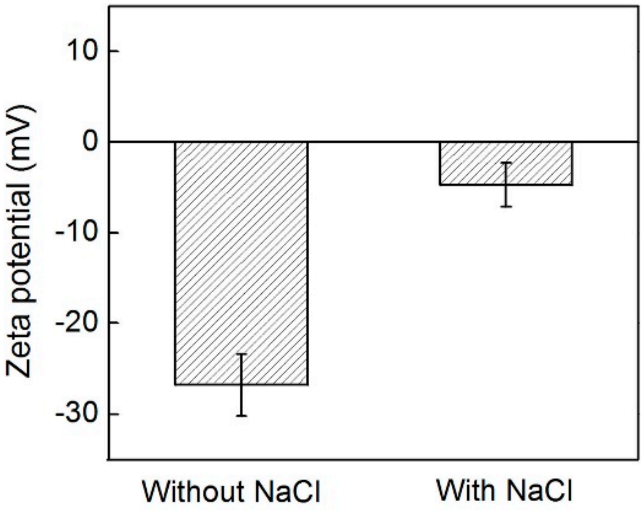

Figure S3. Zeta potential of the emulsions with and without NaCl (1 M).

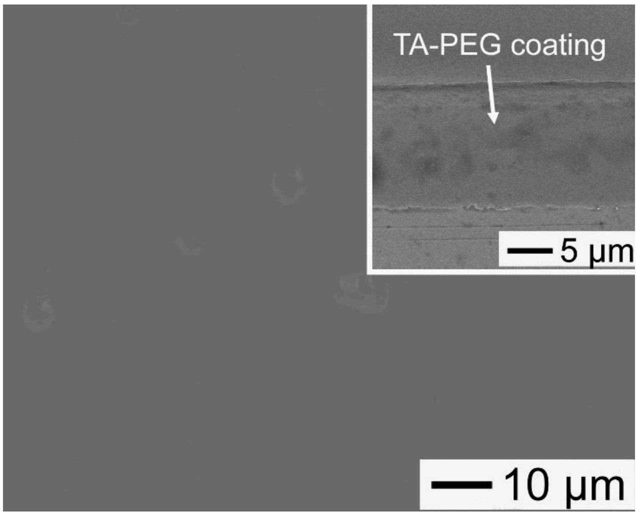

Figure S4. SEM images of the surface morphologies of the PMMA substrates incubated with the TA-PEG emulsion (TA: 5 mg/mL, PEG of M_W =100 kDa: 2.5 mg/mL, NaCl: 1 M) for 120 min. The inset shows a cross-sectional SEM image of the substrate.

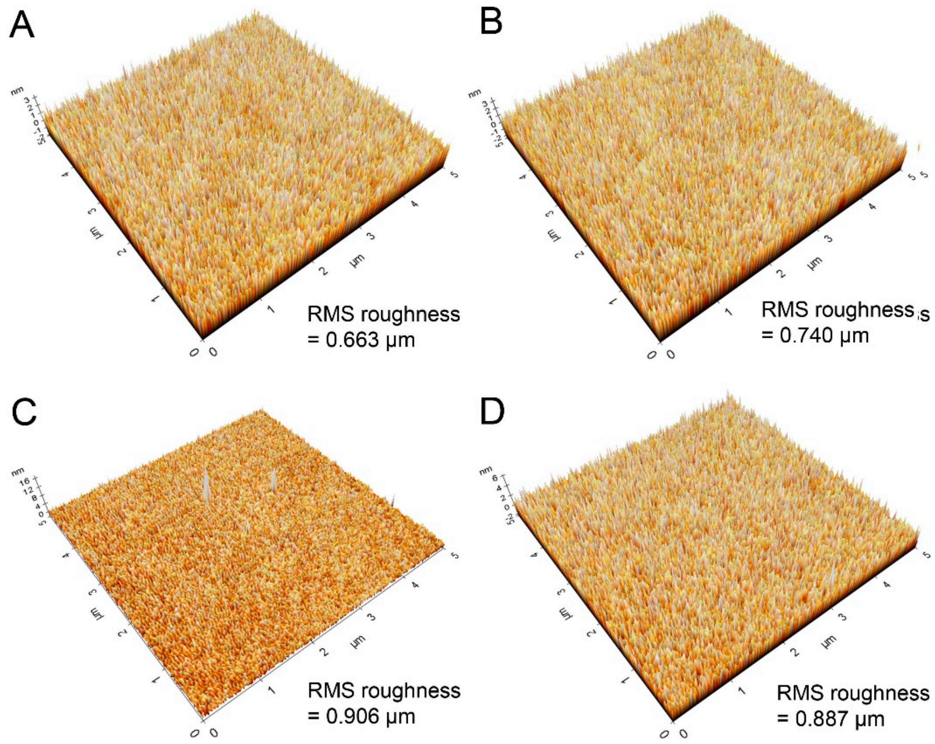

Figure S5. AFM images showing the surface roughness of the PMMA substrate, which were prepared through incubation for 10 min, with the TA-PEG emulsions (PEG of M_W = 100 kDa: 5 mg/mL, NaCl: 2 M) containing different concentrations of TA: (**A**) 2.5 mg/mL, (**B**) 5.0 mg/mL, (**C**) 7.5 mg/mL, and (**D**) 10 mg/mL.

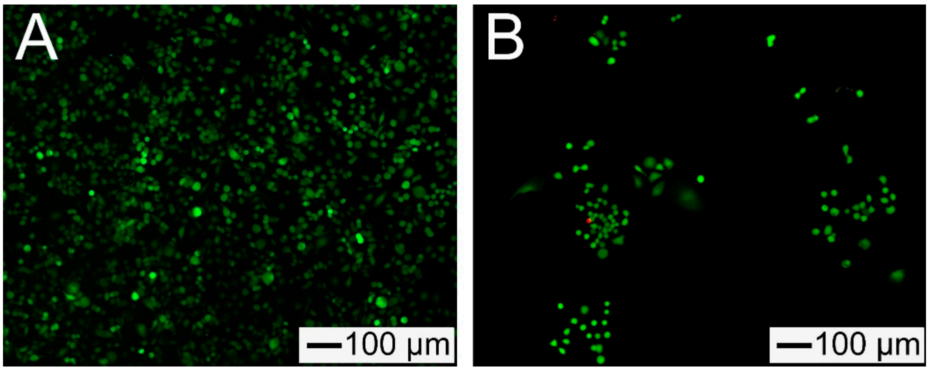

Figure S6. CLS micrographs showing live (green) and dead (red) MC3T3-E1 mouse osteoblast cells on the surface of **(A)** PMMA bare substrate and **(B)** TA-PEG-coated substrate.
